# Efficacy and Safety of Intravenous Magnesium Sulfate in Spinal Surgery: A Systematic Review and Meta-Analysis

**DOI:** 10.3390/jcm13113122

**Published:** 2024-05-26

**Authors:** Jorge Campos, Jose Luis Bas, Claudia Campos, Gonzalo Mariscal, Teresa Bas, Paloma Bas

**Affiliations:** 1 Spine Unit, Department of Orthopedic Surgery and Traumatology, La Fe University and Polytechnic Hospital, 46026 Valencia, Spain; jorcambas@gmail.com (J.C.); pepobh@gmail.com (J.L.B.); teresabas@gmail.com (T.B.); palobasher@gmail.com (P.B.); 2 Son Espases University Hospital, 07120 Palma de Mallorca, Spain; camposbasclaudia@gmail.com

**Keywords:** magnesium sulfate, spinal surgery, multimodal, pain, opioid consumption, meta-analysis

## Abstract

Optimizing pain management in spinal surgery is crucial for preventing adverse events due to delayed mobilization. Magnesium sulfate has potential benefits in spinal surgery because of its analgesic properties and modulation of neurotransmitters and autonomic nervous system. Existing evidence regarding the use of magnesium sulfate is partial and controversial, necessitating a comprehensive meta-analysis to evaluate its efficacy and safety. The aim of this study was to conduct a comprehensive meta-analysis to evaluate the efficacy and safety of magnesium sulfate in spinal surgery compared to other available options. This meta-analysis adhered to the PRISMA guidelines. Patients undergoing spinal surgery were included, with the intervention group receiving intravenous magnesium sulfate (MS) at various doses or combinations, whereas the comparison group received other alternatives or a placebo. The efficacy and safety outcomes were assessed. Data were collected from multiple databases and analyzed using Review Manager version 5.4. Heterogeneity was assessed and fixed- or random-effects models were applied. The meta-analysis included eight studies (*n* = 541). Magnesium sulfate demonstrated significant reductions in pain at 24 h (MD −0.20, 95% CI: −0.39 to −0.02) and opioid consumption (SMD −0.66, 95% CI: −0.95 to −0.38) compared to placebo. Additionally, a decrease in the use of muscle relaxants (SMD −0.91, 95% CI: −1.65 to −0.17) and remifentanil (SMD −1.52, 95% CI: −1.98 to −1.05) was observed. In contrast, an increase in extubation time (MD 2.42, 95% CI: 1.14 to 3.71) and verbal response (MD 1.85, 95% CI: 1.13 to 2.58) was observed compared to dexmedetomidine. In conclusion, magnesium sulfate administration in spinal surgery reduced pain and opioid consumption, and prolonged orientation and verbal response. No significant differences in blood pressure or heart rate were observed between the groups.

## 1. Introduction

Chronic low back pain is a highly prevalent and impactful condition that poses a significant burden to the healthcare system [1]. Its management requires a gradual and progressive approach, prioritizing noninvasive options such as lifestyle changes and physiotherapy. However, in more severe and refractory cases, invasive interventions such as steroid injections or surgical procedures may be necessary [2]. Although steroid injections have shown Level I evidence in relieving radicular pain due to disc herniation, their safety raises concerns due to the rare but serious neurological complications associated with them [3]. In this context, different treatment options for lower back pain have been explored, including the use of products such as ozone, which has shown pain improvement but remains controversial due to short-term assessments and lack of a well-defined mechanism of action [4]. Ozone acts on substances in the body that cause inflammation and pain, such as TNF-α, IL-6, and IL-1β. Ozone alleviates pain by reducing the levels of these substances. In addition, ozone affects enzymes and factors involved in pain pathways, contributing to the regulation of the body’s natural pain relief mechanisms. Furthermore, the importance of reducing opioid consumption and adopting a multimodal approach for pain management has been widely recognized [5].

In the specific context of spinal surgery, particularly in adolescents undergoing spinal fusion procedures for idiopathic scoliosis, extensive research has been conducted on the use of various adjuvant drugs such as ketamine and gabapentin. The primary objective of these studies was to investigate their efficacy in reducing postoperative pain and minimizing the need for opioid medication. Encouragingly, these investigations have demonstrated a significant reduction in opioid consumption within the first 48 h post-surgery, accompanied by a decrease in pain intensity. Moreover, the use of ketamine and gabapentin has shown a favorable safety profile with minimal occurrence of adverse events [6,7]. However, optimizing pain management remains crucial because a delay in mobilization can lead to significant adverse events [8].

Magnesium sulfate (MS) has been extensively studied in spinal surgery because of its potential benefits, including analgesic and antiarrhythmic effects [9]. MS acts as a cofactor in various enzymatic reactions, and plays a key role in the synthesis and release of neurotransmitters involved in mood modulation and pain perception. The impact of magnesium on surgical bleeding is still debated, with some studies showing that it can prolong bleeding time, whereas others suggest that it has a vasodilatory effect on peripheral vessels, leading to hypotension and reduced blood loss [9]. Magnesium sulfate, in particular, has garnered attention in pain management, with intravenous administration proving to be effective for postoperative pain [10]. Magnesium sulfate acts as a physiological antagonist of the N-methyl-D-aspartate (NMDA) receptor, making it useful in various clinical situations such as tachyarrhythmia, ischemia, asthma, spasmophilia, pre-eclampsia, tocolysis, and post-anesthesia shivering [11]. Its effects on postoperative pain relief and opioid consumption have been investigated in gynecological, ophthalmic, and arthroscopic knee surgeries [12]. The precise mechanism underlying the analgesic effects of magnesium is not fully understood; however, it is believed to involve interference with calcium channels and NMDA receptors [12].

Existing evidence regarding the use of magnesium sulfate in spinal surgery is incomplete and controversial. While some studies have found promising results, such as improvement in pain scores and patient satisfaction, reduction in intraoperative bleeding, and improved hemostatic parameters [13,14,15], adverse effects such as prolonged extubation time and slower recovery have also been reported [16]. Despite the partial and controversial evidence on the use of magnesium sulfate in spinal surgery, no meta-analysis specifically focusing on this area has been conducted so far. Tsaousi et al. reported that the existing literature indicates a “low statistical power” in the conducted studies [17], emphasizing the need for a comprehensive analysis of the available data to quantify the efficacy and safety of magnesium sulfate in spinal surgery. Therefore, this study aimed to conduct a thorough meta-analysis to evaluate the efficacy and safety of magnesium sulfate in spinal surgery.

## 2. Materials and Methods

### 2.1. Eligibility Criteria

**Figure 1:**
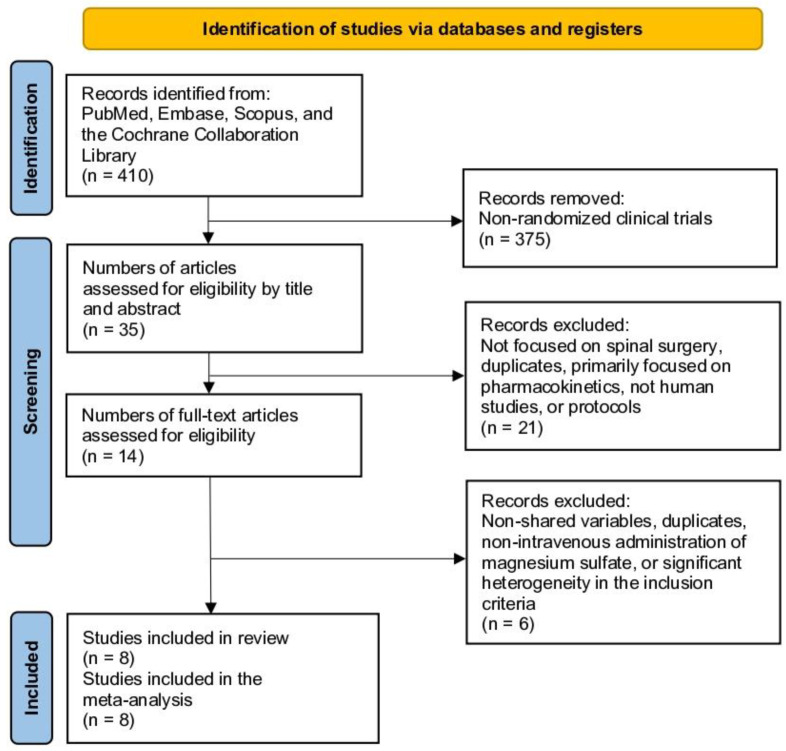
Flow diagram depicting the study selection process (Preferred Reporting Items for Systematic Reviews and Meta-analyses).

This meta-analysis followed the Preferred Reporting Items for Systematic Reviews and Meta-Analyses (PRISMA) guidelines (Figure 1) [18]. Inclusion criteria were established based on the PICOS framework. This study included adult patients who underwent spinal surgery. The intervention group comprised patients treated with various doses or combinations of intravenous magnesium sulfate (MS). The comparison group comprised patients treated with other alternatives or a placebo. The outcomes of interest included efficacy measures such as pain control, drug consumption, and hemodynamic outcomes, as well as safety results assessed through the evaluation of complications. The studies of interest were randomized controlled trials.

Duplicate studies were excluded to prevent the inclusion of redundant data and potential bias. Non-randomized studies were excluded because of their inherent limitations in controlling confounding factors and establishing causal relationships. Reviews were excluded because they provided a summary of the existing literature rather than primary data. Studies with incomplete or missing data were excluded to maintain the integrity and reliability of the analyses. Finally, studies that did not share variables in at least two included studies were excluded to enable meaningful pooling of data and facilitate robust statistical analysis. 

### 2.2. Information Sources and Search Methods for Identification of Studies

A systematic search was conducted in multiple databases, including PubMed, Embase, Scopus, and Cochrane Collaboration Library, without limitations on publication date or language. The search MESH terms used were “magnesium” and “spine” to identify relevant studies (Supplementary File S1). In addition to the database search, the reference lists of the included studies were thoroughly reviewed to identify additional relevant studies. Two independent reviewers with level V experts according to Tang et al. [19] screened all the titles and abstracts for eligibility. In case of persistent disagreement, a third reviewer was consulted to reach a consensus.

### 2.3. Data Extraction and Data Items

Two independent reviewers with level V experts according to Tang et al. [19] were involved in the assessment process, and in cases of disagreement, a third reviewer was consulted to reach consensus. The baseline characteristics of the included studies, such as study name, region, study period, number of patients, average age, number of female participants, etiology, and dosage of spinal medication, were collected. The main outcomes assessed in the meta-analysis included pain levels measured using the Visual Analog Scale (VAS), medication consumption (opioids, muscle relaxants, and remifentanil), hemodynamic measurements, including mean arterial pressure (MAP) and heart rate (HR), extubation time, response to verbal commands, orientation time, and adverse events. 

### 2.4. Assessment of Risk of Bias in Included Studies

**Figure 2:**
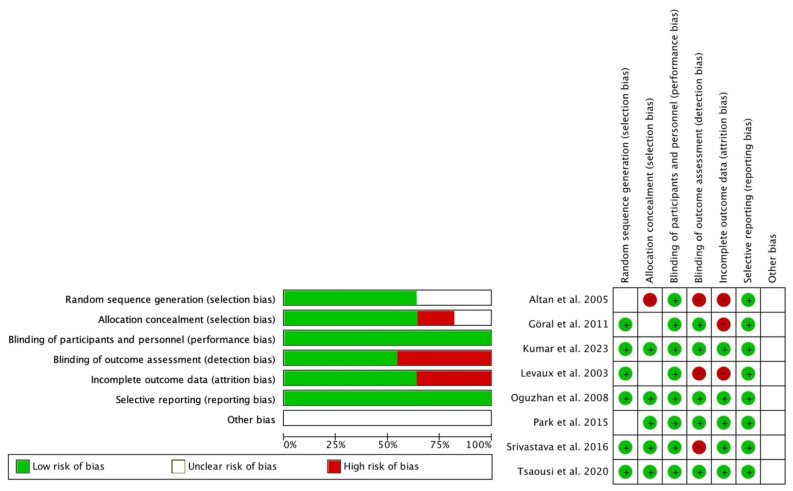
Assessment of the risk of bias (green = low risk; red = high risk; white = unknown) [9,10,14,15,16,20,21,22].

To assess the risk of bias within the included studies, the Cochrane Collaboration’s risk of bias tool was utilized, and the analysis was performed using the Review Manager software (Figure 2). Six domains were evaluated: random sequence generation, allocation concealment, blinding of participants and personnel, blinding of outcome assessments, incomplete outcome data, and selective reporting.

### 2.5. Assessment of Results

Review Manager 5.4 software was utilized for data analysis. For continuous outcomes, mean differences were calculated and odds ratios were calculated for dichotomous outcomes. The results were presented with 95% confidence intervals to indicate the precision of the estimates. Heterogeneity was assessed using the Chi^2^ and I^2^ tests, with I^2^ values greater than 25%, 50%, and 75% indicating low, moderate, and high heterogeneity, respectively. A fixed-effects model was used when no significant heterogeneity was detected, whereas a random-effects model was used when heterogeneity was present. To obtain data from figures in the articles, the WebPlotDigitizer version 4.5 was utilized. Missing data were handled according to the guidelines provided in the Cochrane Handbook [23]. 

### 2.6. Risk of Bias across the Studies

Publication bias was assessed using Review Manager software. One method commonly used to visually inspect publication bias is funnel plots. A funnel plot is a scatter plot that displays the effect sizes of individual studies on the horizontal axis, and a measure of study precision (such as the standard error or sample size) on the vertical axis. In the presence of publication bias, smaller studies with non-significant results may be missing from the plot, resulting in an asymmetrical shape. 

### 2.7. Additional Analyses

Subgroup analyses were performed, considering the different time points of outcome measurement, which allowed for a more detailed examination of the results at specific intervals.

Sensitivity analyses were conducted to compare pain management therapy with different control groups including placebo, clonidine, dexamethasone, and dexmedetomidine.

Additionally, a Grading of Recommendations Assessment, Development, and Evaluation (GRADE) assessment was conducted using the GRADEpro tool [20]. The GRADE approach involves evaluating the quality of evidence and the strength of recommendations, considering factors such as the risk of bias, heterogeneity, precision of results, and consistency across studies.

## 3. Results

### 3.1. Study Selection

The initial database search yielded 410 studies. After filtering for clinical trials, 375 studies were eliminated, leaving a total of 35 studies. Upon examining the titles and abstracts of these 35 studies, 21 were excluded because they did not focus on spinal surgery, were primarily centered on pharmacokinetics, were not human studies, or were protocols. After reviewing the full text of the remaining 14 articles, 6 were removed due to non-shared variables, duplicates, non-intravenous administration of MS, or significant heterogeneity in the inclusion criteria, resulting in a total of 8 articles. No additional articles were included in the review of references of the included studies. Finally, eight articles were included in the meta-analysis (Figure 1) [9,10,14,15,16,20,21,22].

### 3.2. Study Characteristics

Table 1 summarizes the main characteristics of the included studies. A total of 8 studies with 541 patients were included (245 in the MS group, 143 in the placebo group, 93 in the dexamethasone group, and 60 in the dexmedetomidine group). The mean age ranged from 35.2 to 55.9 years, with 238 of 541 (50%) being female. The etiology of the indication for surgery, as well as the dosing regimen for MS, are shown in Table 1. The risk of bias in these studies is shown in Figure 2.

### 3.3. Pain

**Figure 3:**
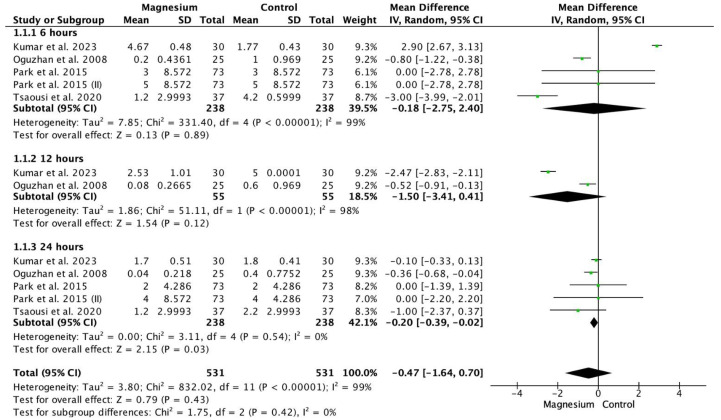
Forest plot displaying pain measured using Visual Analog Scale (VAS) [10,16,20,21].

There were no significant differences in the global VAS (MD −0.47, 95% CI −1.64 to 0.70; participants = 1062; studies = 12; I^2^ = 99%) (Figure 3). Similarly, no significant differences were found at 6 h (MD −0.18, 95% CI −2.75 to 2.40; participants = 476; studies = 5; I^2^ = 99%) and 12 h (MD −1.50, 95% CI −3.41 to 0.41; participants = 110; studies = 2; I^2^ = 98%). However, at 24 h, the magnesium group showed significantly lower pain than the control group (MD −0.20, 95% CI −0.39, −0.02; participants, 476; studies, 5; I^2^ = 0%). When comparing MS versus placebo, MS demonstrated a greater significant reduction in global VAS (MD −0.99, 95% CI −1.59 to −0.39; participants = 298; studies = 12; I^2^ = 85%) at 12 h (MD −0.52, 95% CI −0.91 to −0.13; participants = 50; studies = 2; I^2^ = 0%) and 24 h (MD −0.39, 95% CI −0.70 to −0.08; participants = 124; studies = 5; I^2^ = 0%). No significant differences were found when comparing MS with dexmedetomidine (MD 0.11, 95% CI −2.75 to 2.98; participants = 180; studies = 12; I^2^ = 100%). Similarly, no significant differences were observed when comparing MS with dexamethasone (MD 0.00, 95% CI −1.01 to 1.01; participants = 584; studies = 12; I^2^ = 0%).

### 3.4. Drug Consumption

**Figure 4:**
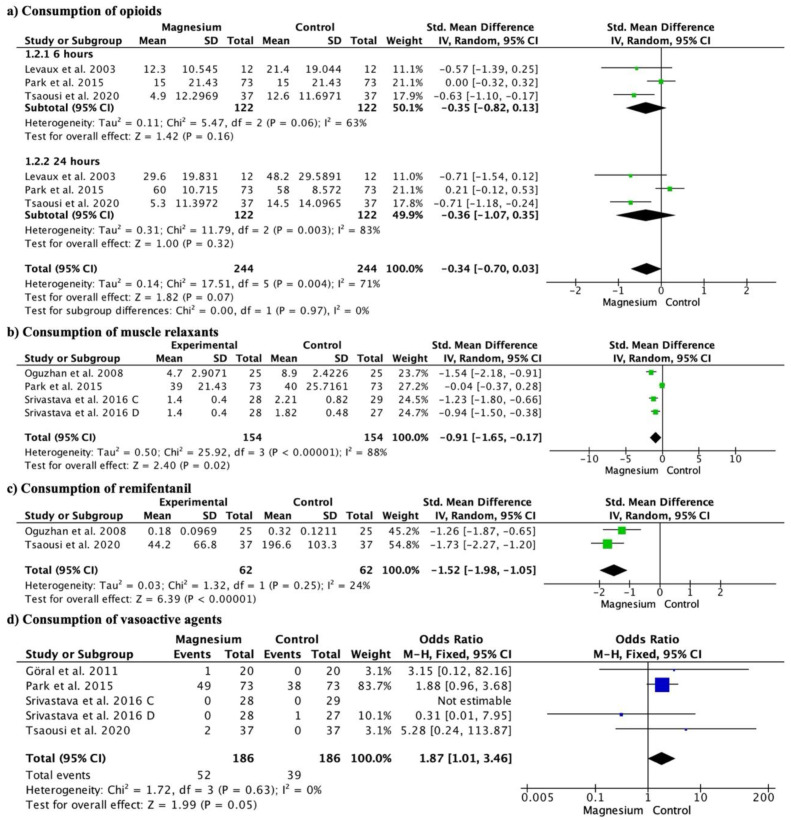
Forest plot illustrating opioid consumption (**a**), muscle relaxant consumption (**b**), remifentanil consumption (**c**), and vasopressor consumption (**d**) [9,10,14,16,20,21,22].

Regarding the consumption of opioids, there were no significant differences (SMD −0.34, 95% CI −0.70 to 0.03; participants = 488; studies = 6; I^2^ = 71%) (Figure 4a), at 6 h (SMD −0.35, 95% CI −0.82 to 0.13; participants = 244; studies = 3; I^2^ = 63%), or at 24 h (SMD −0.36, 95% CI −1.07 to 0.35; participants = 244; studies = 3; I^2^ = 83%). When comparing MS versus placebo, magnesium significantly reduced opioid consumption (SMD −0.66, 95% CI −0.95 to −0.38; participants = 196; studies = 6; I^2^ = 0%), both at 6 h (SMD −0.62, 95% CI −1.03 to −0.21; participants = 98; studies = 3; I^2^ = 0%) and 24 h (SMD −0.71, 95% CI −1.12 to −0.30; participants = 98; studies = 3; I^2^ = 0%). There were no significant differences when comparing MS with dexamethasone (SMD 0.10, 95% CI −0.13 to 0.33; participants = 292; studies = 6; I^2^ = 0%) overall or at 6 or 24 h.

Consumption of muscle relaxants was significantly lower in the MS group (SMD −0.91, 95% CI −1.65 to −0.17; participants = 308; studies = 4; I^2^ = 88%) (Figure 4b). MS showed significantly lower consumption of muscle relaxants than placebo (SMD −1.54, 95% CI −2.18 to −0.91; participants = 50; studies = 4; I^2^ = 0%) and dexmedetomidine (SMD −1.54, 95% CI −2.18 to −0.91; participants = 50; studies = 4; I^2^ = 0%). There were no significant differences with respect to dexamethasone (SMD −1.54, 95% CI −2.18, −0.91; participants = 50; studies = 4; I^2^ = 0%).

MS also showed a significantly lower consumption of remifentanil (SMD −1.52, 95% CI −1.98 to −1.05; participants = 124; studies = 2; I^2^ = 24%) (Figure 4c).

There were no significant differences in the use of vasoactive agents (OR 1.87, 95% CI 1.01 to 3.46; participants = 372; studies = 5; I^2^ = 0%) (Figure 4d). Neither when compared to placebo (OR 4.22, 95% CI 0.46 to 38.99; participants = 114; studies = 5; I^2^ = 0%), dexamethasone (OR 1.88, 95% CI 0.96 to 3.68; participants = 146; studies = 5; I^2^ = 0%), nor dexmedetomidine (OR 0.31, 95% CI 0.01 to 7.95; participants = 112; studies = 5; I^2^ = 0%).

### 3.5. Hemodynamics

**Figure 5:**
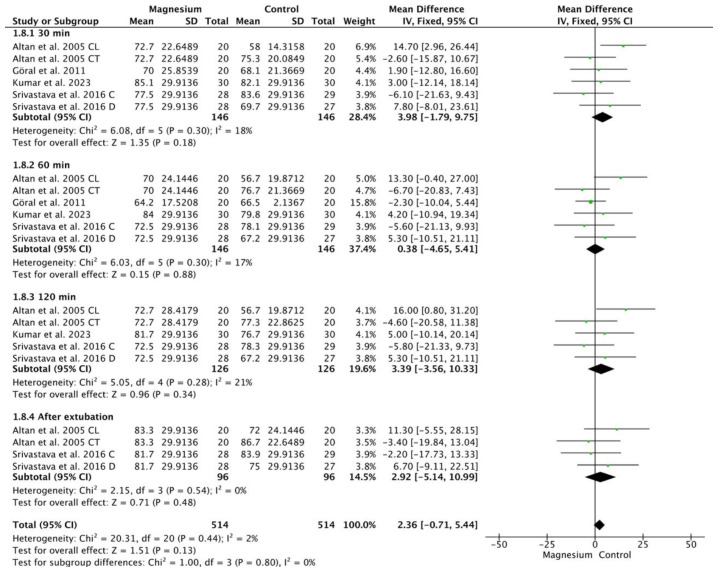
Forest plot presenting the results of heart rate at 30 min, 60 min, 120 min, and after extubation [9,15,20,22].

There were no significant differences between the groups regarding global heart rate (MD 2.36, 95% CI −0.71 to 5.44; participants = 1028; studies = 21; I^2^ = 2%) (Figure 5), nor in any of the subgroups based on follow-up time. Compared to the placebo, the MS group showed a significantly higher heart rate (MD 6.15, 95% CI 1.26 to 11.04; participants = 240; studies = 21; I^2^ = 50%). There were no significant differences when comparing MS with clonidine (MD −4.31, 95% CI −11.70 to 3.08; participants = 160; studies = 21; I^2^ = 0%) or with dexmedetomidine (MD 1.58, 95% CI −3.10 to 6.26; participants = 628; studies = 21; I^2^ = 0%).

**Figure 6:**
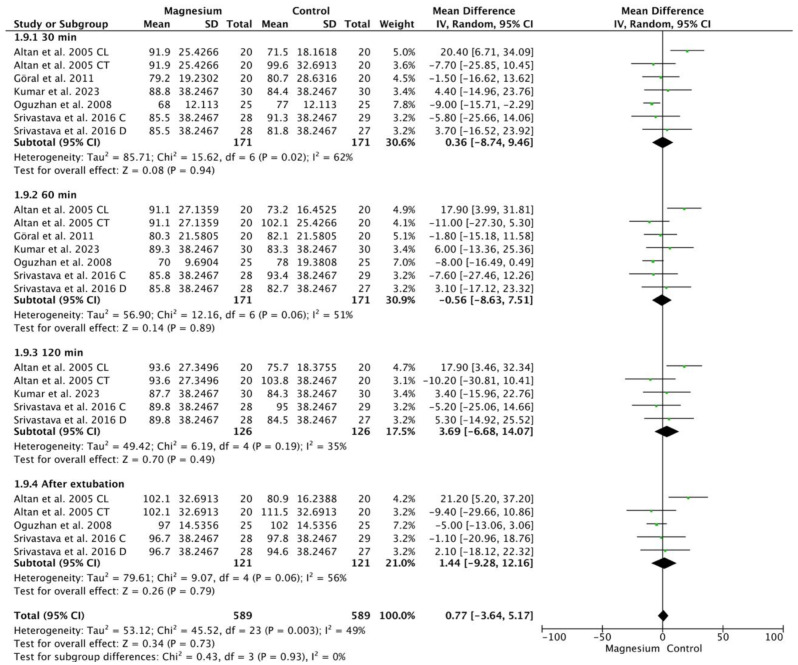
Forest plot showing the mean arterial pressure at 30 min, 60 min, 120 min, and after extubation [9,10,15,20,22].

Mean arterial pressure (MAP) did not show significant differences between the groups (MD 0.77, 95% CI −3.64 5.17; participants = 1178; studies = 24; I^2^ = 49%) (Figure 6). There were no significant differences compared with the placebo group (MD 4.62, 95% CI −3.66 to 12.89; participants = 390; studies = 24; I^2^ = 80%). Compared to clonidine, MS showed a significantly lower MAP (MD −9.64, 95% CI −18.93–−0.35; participants = 160; studies = 24; I^2^ = 0%). There were no significant differences with respect to dexmedetomidine (MD 0.77, 95% CI −5.21, 6.76; participants, 628; studies, 24; I^2^ = 0%).

### 3.6. Extubation Time, Response to Verbal Commands and Orientation Time

**Figure 7:**
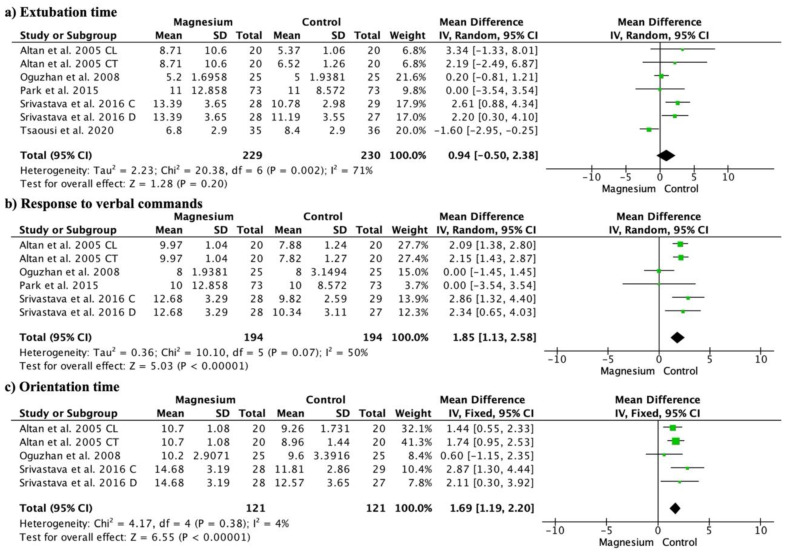
Forest plot demonstrating extubation time (**a**), response to verbal commands (**b**), and orientation time (**c**) [10,15,16,21,22].

Extubation time was not significantly different between the groups (MD 0.94, 95% CI −0.50 to 2.38; participants = 459; studies = 7; I^2^ = 71%) (Figure 7a). There were no significant differences compared to the placebo group (MD −0.17, 95% CI −2.01 to 1.66; participants = 161; studies = 7; I^2^ = 71%), dexamethasone group (MD 0.00, 95% CI −3.54 to 3.54; participants = 146; studies = 7; I^2^ = 0%), or clonidine group (MD 2.77, 95% CI −0.54 to 6.07; participants = 80; studies = 7; I^2^ = 0%). Compared with dexmedetomidine, the MS group showed a significantly longer extubation time (MD: 2.42, 95% CI 1.14 to 3.71; participants = 112; studies = 7; I^2^ = 0%).

The response to verbal commands was significantly higher in the MS group (MD 1.85, 95% CI 1.13 to 2.58; participants = 388; studies = 6; I^2^ = 50%) (Figure 7b). There were no significant differences compared to the placebo group (MD 1.14, 95% CI −0.89 to 3.18; participants = 90; studies = 6; I^2^ = 84%), or the dexamethasone group (MD 0.00, 95% CI −3.54 to 3.54; participants = 146; studies = 6; I^2^ = 0%). However, the MS group showed a significantly longer response time to verbal commands than the clonidine group (MD 2.12, 95% CI 1.61 to 2.62; participants = 80; studies = 6; I^2^ = 0%) and dexmedetomidine group (MD 2.62, 95% CI 1.49 to 3.76; participants = 112; studies = 6; I^2^ = 0%).

Orientation time was also significantly longer in the MS group (MD: 1.69, 95% CI 1.19 to 2.20; participants = 242; studies = 5; I^2^ = 4%) (Figure 7c). The orientation time was significantly longer in the MS group compared to placebo (MD 1.27, 95% CI 0.47 to 2.06; participants = 90; studies = 5; I^2^ = 0%), clonidine (MD 1.74, 95% CI 0.95 to 2.53; participants = 40; studies = 5; I^2^ = 100%), and dexmedetomidine (MD 2.54, 95% CI 1.35 to 3.73; participants = 112; studies = 5; I^2^ = 0%).

### 3.7. Adverse Events

The adverse events are shown in Table 2. Hypotension was significantly higher in the MS group (OR 1.97, 95% CI 1.10 to 3.52; participants = 396; studies = 6; I^2^ = 0%). There were no significant differences between MS and placebo (OR 2.71, 95% CI 0.74 to 9.95; participants = 138; studies = 6; I^2^ = 0%) or dexmedetomidine (OR 0.18, 95% CI 0.01 to 3.91; participants = 112; studies = 6; I^2^ = 0%). However, dexamethasone showed significantly lower hypotension than MS (OR 2.19, 95% CI 1.10 to 4.36; participants = 146; studies = 6; I^2^ = 0%).

There were no significant differences in the incidence of postoperative nausea and vomiting (PONV) (OR 0.74, 95% CI 0.44 to 1.23; participants = 294; studies = 4; I^2^ = 38%). MS showed significantly lower PONV incidence than placebo (OR 0.37, 95% CI 0.16 to 0.85; participants = 148; studies = 4; I^2^ = 0%). There were no significant differences compared with dexamethasone (OR 1.20, 95% CI 0.60 to 2.39; participants = 146; studies = 4; I^2^ = 0%).

There were no significant differences in the incidence of shivering (OR 0.24, 95% CI 0.01 to 6.80; participants = 98; studies = 2; I^2^ = 74%) compared to placebo. There were no significant differences in the incidence of arrhythmia (OR 1.62, 95% CI 0.19 to 13.63; participants = 186; studies = 3; I^2^ = 0%) compared to dexmedetomidine. No other comparisons were performed.

### 3.8. Publication Bias

The assessment of publication bias through visual inspection of the funnel plots revealed a high level of publication bias due to the asymmetry observed in the majority of variables. Several key variables that display this asymmetry are shown in Supplementary Figure S1.

### 3.9. GRADE

Table 3 presents the GRADE assessments. There was low certainty for the VAS variable, high certainty for the heart rate, and moderate certainty for the remaining variables. The studies exhibited a high risk of publication bias; the effect size was not large, and in some cases, the results showed considerable variability.

## 4. Discussion

The reduction in pain observed with magnesium compared to placebo could be attributed to its neuroprotective and anti-inflammatory effects, as suggested by Hassan et al. [24]. Magnesium promotes neuroplasticity and exerts a neuroprotective effect in postoperative cognitive dysfunction and neuronal degeneration [24]. Additionally, magnesium eliminates hydrogen peroxide (H_2_O_2_) and releases hydrogen (H_2_) to neutralize harmful reactive oxygen species (ROS), resulting in significant antioxidant and anti-inflammatory effects in the treatment of intervertebral disc degeneration (IVDD) [24,25]. Moreover, magnesium has the potential to attenuate the elevation of S100B protein, an indicator of oxidative stress and a precursor of amyloid [25]. While the comparison of patient-reported outcome measures (PROMs) was limited to the Visual Analog Scale (VAS), individual studies have observed that satisfaction with magnesium may also be attributed to its effect on postoperative sore throat, which has been shown to be similar to that of corticosteroids [21]. This finding is particularly intriguing considering the adverse events associated with corticosteroids, such as their negative effects on glucose metabolism. The anti-inflammatory effects of magnesium may explain this phenomenon. By reducing pain with magnesium, the risk of nausea and vomiting is likely reduced, resulting in a lower incidence of postoperative sore throat [26,27]. However, it is important to note that the minimal clinically important difference (MCID) was not evaluated in any of the studies, highlighting the need for future research to address this aspect.

In addition, magnesium has been shown to significantly reduce opioid consumption compared to placebo. This is attributed to the protective effect of magnesium in the brain, as it non-competitively blocks NMDA receptors, thereby reducing glutamate release and preventing excessive calcium entry into neuronal cells. This mechanism helps preserve cell integrity and provides neuroprotection [12]. Other drugs that act as NMDA receptor antagonists, such as ketamine and gabapentin, have also been shown to decrease opioid consumption [12]. Furthermore, owing to its neuroprotective effects, magnesium sulfate has been recommended to reduce the risk of cerebral palsy in preterm infants, especially those at risk of delivery before 32 weeks of gestation [28]. Intracisternal magnesium sulfate reduces the incidence of cerebral vasospasm and delayed cerebral ischemia by acting as a vasodilator and stabilizing neuronal functions. Additional intravenous hydrogen therapy can provide antioxidant effects that further improve the clinical outcomes and reduce oxidative stress in the brain [29].

In terms of hemodynamics, the magnesium group exhibited a lower mean arterial pressure than the corticosteroid group did. This can be attributed to the hypotensive effect of magnesium, which is mediated through its vasodilatory properties. Magnesium has a similar effect as calcium channel-blocking medications, relaxing blood vessels, and reducing blood pressure. It also competes with sodium in the muscle cells of the blood vessels, contributing to a reduction in blood pressure. Additionally, magnesium has beneficial effects on endothelial function and the inner layer of blood vessels, improving its ability to dilate and reduce inflammation [30].

This vasodilation may explain the higher heart rate observed in the present meta-analysis. On the other hand, corticosteroids are known to stimulate the adrenergic system, which could explain the higher mean arterial pressure in the corticosteroid group. The hypotensive effect of magnesium has been suggested to explain reduced bleeding during surgery in some studies [30]. Furthermore, magnesium blocks catecholamines, inhibiting the sympathetic nervous system [31]. Regarding coagulation, a study reported the results and found that the activated partial thromboplastin time was prolonged in the magnesium group, whereas other coagulation parameters showed no significant differences between the two groups [9].

Furthermore, magnesium has been shown to lower the consumption of muscle relaxants. Magnesium sulfate inhibits the response of acetylcholine receptors in muscle cells and enhances the action of the muscle relaxant, vecuronium. This could improve muscle relaxation during medical procedures, without the need for higher doses of muscle relaxants. However, further studies are required to confirm these clinical advantages. One possible drawback is the need for careful adjustment of magnesium sulfate and vecuronium doses to avoid adverse effects or excessive muscle relaxation [32].

Therefore, it is important to consider the potential for excess Mg. Magnesium toxicity is generally considered when levels exceed 4 to 5 mmol/L (9.7 to 12.2 mg/dL), which can be associated with adverse effects. Hypermagnesemia, characterized by high levels of magnesium in the blood, can present with symptoms such as nausea, dizziness, confusion, and weakness, and is associated with an increased risk of mortality in hospitalized patients. The prevalence of hypermagnesemia in clinical settings varies, with rates of 3.0% in the general population and 5.7–9.3% in hospitals. Timely identification and management are crucial to prevent serious complications, especially in patients with renal insufficiency [33].

In contrast, the recovery time, particularly measured by response to verbal commands and orientation time, was longer in the magnesium group. This could be related to the decrease in blood pressure and demonstrated muscle relaxant effect observed in the variable of lower muscle relaxant consumption. The hypotensive effect of Mg, which contributes to its vasodilatory properties, may result in a decrease in cerebral perfusion and oxygenation, leading to a longer recovery time and delayed cognitive function. Additionally, the muscle relaxant effect of magnesium could also contribute to prolonged recovery time, as excessive muscle relaxation may affect the patient’s ability to respond to verbal commands and regain orientation.

The other comparator used in this meta-analysis was dexmedetomidine. Dexmedetomidine is an alpha-2 adrenoceptor agonist that attenuates surgical stress. Dexmedetomidine reduced the consumption of propofol and fentanyl more than magnesium. Srivastav et al. observed a greater improvement in hemodynamic stability with dexmedetomidine. Dexmedetomidine has higher affinity and selectivity for α2-adrenergic receptors, which gives it greater efficacy in reducing opioid and propofol consumption, as well as a better hemodynamic profile compared to magnesium. However, it has a higher incidence of hypotension and bradycardia as adverse events than magnesium [22]. Kumar also observed better pain control with dexmedetomidine than with magnesium combined with ropivacaine. This meta-analysis did not reveal any significant differences in pain. Additionally, the dexmedetomidine group exhibited a higher consumption of muscle relaxants. Dexmedetomidine showed a shorter response time to verbal commands and orientation time compared to placebo. No significant differences were observed for the remaining variables. It is important to note that dexmedetomidine, an alpha-2 adrenoceptor agonist, can prolong the effect of local anesthesia owing to its vasoconstrictive effects [34]. A recent meta-analysis on dexmedetomidine in spinal surgery found that it increases the risk of intraoperative hypotension and bradycardia, particularly when a loading dose is administered and total intravenous anesthesia is used. There was a significant increase in the risk of bradycardia in the inhalation anesthesia group. Overall, no significant reduction in intraoperative blood loss was observed; however, a significant decrease in blood loss was noted in the total inhalation anesthesia subgroup [35].

Furthermore, magnesium can be used in combination with other drugs or in pain control strategies to achieve synergistic or additive effects. Therefore, although the differences with corticosteroids may have been more moderate, this could also be attributed to the small sample size of comparative studies between magnesium sulfate and corticosteroids or the short follow-up time during which measurements were taken, often limited to 24 h in many cases.

Combining magnesium with other analgesic medications or techniques, such as opioids, local anesthetics, or regional anesthesia, has been shown to enhance pain relief and improve patient outcomes. The synergistic or additive effects of magnesium with these interventions can provide a more comprehensive and effective approach for pain management.

It is important to note that the duration and timing of measurements and follow-up in clinical studies can influence the differences observed between magnesium and corticosteroids. Long-term follow-up and assessment beyond the immediate postoperative period may be necessary to fully capture the potential benefits of magnesium in pain control and recovery.

Regarding the administration method, this study only included intravenous administration. Only one study has analyzed the effects of transforaminal magnesium compared to ozone. Both in the short term (2 weeks) and in the long term (3 months), transforaminal magnesium showed improvement in pain measured using the Visual Analog Scale (VAS) and functionality measured by the Oswestry Disability Index (ODI) compared to ozone.

There is no consensus on whether to use a bolus, bolus plus infusion, or infusion only [16]. In this meta-analysis, the magnesium protocols varied. The evaluated magnesium administration protocols included both the loading doses and maintenance infusions. The loading doses were administered intravenously immediately prior to anesthesia induction, ranging from 30 to 50 mg/kg diluted in saline, and infused over periods of 10 to 30 min. The maintenance infusion was administered continuously during the surgical procedure at a rate of 10–20 mg/kg/h. One study compared the infiltration of ropivacaine with 500 mg magnesium versus ropivacaine alone. In summary, the protocols consisted of administering an intravenous loading dose of 30–50 mg/kg prior to the intervention, followed by a continuous magnesium infusion of 10–20 mg/kg/h during the surgical procedure.

Although formal tests to evaluate the different magnesium administration strategies could not be carried out, visual inspection of the forest plots did not seem to show any relationship, although this is a visual interpretation and should be interpreted with caution.

In other types of surgery, such as cardiac surgery, magnesium reduced the occurrence of postoperative atrial fibrillation, especially within the first 24 h. It also decreases ventricular arrhythmias without increasing adverse effects [36]. Intra-articular magnesium reduced postoperative pain following arthroscopic surgery compared to placebo, with no difference compared to bupivacaine. The combination of magnesium and bupivacaine provided greater pain relief and delayed the request for the first analgesic compared with bupivacaine alone, without additional adverse effects. Furthermore, experimental studies have suggested that Mg has a protective effect on cartilage and chondrocytes [37]. A meta-analysis found that magnesium significantly reduced morphine consumption at 24 h, delayed the first analgesic request, and decreased shivering without altering postoperative pain, bradycardia, or nausea and vomiting [38]. Compared to another meta-analysis on magnesium for different indications, our study included a greater number of variables, such as the consumption of muscle relaxants, which was lower in the magnesium group, as well as the consumption of remifentanil.

Regarding the influence of the specific type of surgery, limited information was available from these studies. Among those that focused on laminectomies [16,20], Tsaousi et al. achieved the greatest pain reduction at 6 and 12 h [16], whereas Kumar et al. demonstrated the most significant pain reduction at 12 h [20]. Tsaousi et al. also reported the highest reduction in opioid and remifentanil consumption, and it was the only study that reported a shorter extubation time in the MS group [16]. In the study conducted by Göral et al. [9], which utilized microscopic surgery, few variables were shared with other articles, and no significant differences were observed compared to the control group [9]. Another study specifically addressed lumbar disc surgery without providing further details of the specific type [10]. This study also found significantly favorable results for MS in terms of pain reduction and muscle relaxant consumption compared with the control group [10]. The remaining articles could not be visually inspected due to the use of broad inclusion criteria such as “spine surgery” or “lumbar arthrodesis” or “lumbar spine surgery” or “elective spine surgery”.

A meta-analysis compared intrathecal magnesium sulfate in various orthopedic and non-orthopedic surgeries [39]. This study observed that the addition of intrathecal magnesium sulfate to lipophilic opioids and local anesthetics significantly increased the duration of spinal anesthesia, particularly in non-obstetric studies [39]. The onset of sensory and motor blockade was not delayed, and the incidence of hypotension and pruritus was similar between the groups [39]. However, this meta-analysis did not assess pain or consumption of additional drugs. Hemodynamic parameters were not evaluated in this study [39]. Our meta-analysis concurred with previous findings regarding the prolongation of anesthesia duration, with the current analysis focusing on the time of orientation and response to verbal commands, whereas the previous meta-analysis examined the total duration of spinal anesthesia.

### Limitations

This study has some limitations. These included a small number of included studies, which limited the conduct of consistent subgroup analyses and sensitivity analyses, as well as the ability to evaluate the effect of different doses and various surgical indications. Future studies should report results based on the exact type of surgery performed to provide more specific recommendations. Also, publication bias was assessed through visual inspection rather than formal tests, and a small number of articles were available for the control groups in some cases. Difficulties related to missing data were also encountered, although established Cochrane rules were followed. Additionally, some interesting variables, such as patient satisfaction, reduction in intraoperative bleeding, and improved hemostatic parameters, were reported in one study and could not be compared through meta-analysis. Future studies should consider using data on these variables. These limitations should be taken into account when interpreting the results and emphasize the need for further research addressing these limitations to obtain a more comprehensive understanding of the use of magnesium in the management of postoperative pain.

## 5. Conclusions

In summary, this meta-analysis demonstrated that the administration of magnesium sulfate in spinal surgery significantly reduced pain at 24 h and decreased the consumption of opioids, muscle relaxants, and remifentanil compared to placebo and other analgesics. Additionally, magnesium sulfate prolonged orientation and responses to verbal commands. There were no significant differences in the blood pressure or heart rate between the groups. This multimodal approach with magnesium sulfate appeared to provide more effective control of postoperative pain without increasing adverse effects. These findings suggest that magnesium sulfate could enhance optimized recovery protocols following spinal surgery and address existing issues related to opioid consumption. However, the translation of clinical benefits to patient outcomes and their impact on hospital stay, patient satisfaction, and quality of care remain to be determined.

## Figures and Tables

**Table 1:** Baseline characteristics of the included studies.

Study	Region	Period	n MS/CL/Placebo	Age MS/CL/Placebo	n Female MS/CL/Placebo	Etiology	Dose of Magnesium Sulfate
Altan et al., 2005 [15]	Turkey	NR	20/20/20	42.3/40.8/44.9	7/9/7	Spine surgery	30 mg/kg 1 over a 15-min period before induction of anaesthesia and 10 mg/kg 1 h.
Göral et al., 2011 [9]	Turkey	NR	20/NA/20	48.0/NA/49.0	10/NA/11	Single-level microscopic lumbar discectomy	50 mg/kg in 100 mL saline by slow infusion over 10 min, followed by a continuous infusion of 20 mg/kg/h.
Kumar et al., 2023 [20]	India	NR	30/30/NA	35.2/38.2/NA	8/5/NA	Single-level lumbar laminectomy	15 mL of 0.75% ropivacaine + 500 mg equivalent to 1 mL + 4.0 mL normal saline. The total volume of the solution infiltrated was 20 mL in both groups.
Levaux et al., 2003 [14]	Belgium	NR	12/NA/12	55.0/NA/46.0	8/NA/5	Lumbar arthrodesis	50 mg/kg in 250 mL of normal saline over 30 min immediately before induction of anaesthesia.
Oguzhan et al., 2008 [10]	Turkey	2005 to 2006	25/NA/25	44.0/NA/42.0	12/NA/11	Lumbar disc surgery	30 mg/kg (over 10 min) starting immediately after induction of anesthesia and completed before intubation; the infusion was then continued at 10 mg/kg/h throughout surgery.
Park et al., 2015 [21]	Korea	2013 to 2014	73/73/NA	51.0/51.0/NA	32/31/NA	Lumbar spine surgery	30 mg/kg in a total of 100 mL normal saline was given for 10 min before the induction of anesthesia, followed by continuous infusion of at 10 mg/kg/h until the end of operation.
Srivastava et al., 2016 [22]	India	NR	30/30/30	48.3/45.9/46.6	13/12/14	Elective spine surgery	Loading dose 50 mg/kg before induction over a period of 15 min and maintenance 15 mg/kg/h throughout the surgery.
Tsaousi et al., 2020 [16]	Greece	2020	35/NA/36	55.9/49.0	22/NA/21	Lumbar laminectomy	20 mg/kg diluted in isotonic saline to a volume of 100 mL was infused as an intravenous (i.v.) bolus dose over 15 min before anesthesia induction and thereafter 20 mg/kg/h was continuously infused until surgery completion.

NA: Not applicable; NR: Not reported.

**Table 2:** Adverse events.

Effect Size	Control Group	n Participants	Fixed Effect Model (OR 95% CI)	I^2^ (%)
Hypotension	Placebo	138	OR 2.71, 95% CI 0.74 to 9.95	0%
	Dexmedetomidine	112	OR 0.18, 95% CI 0.01 to 3.91	0%
	Dexamethasone	146	OR 2.19, 95% CI 1.10 to 4.36	0%
PONV	Placebo	148	OR 0.37, 95% CI 0.16 to 0.85	0%
	Dexamethasone	146	OR 1.20, 95% CI 0.60 to 2.39	0%
Shivering	Placebo	98	* OR 0.24, 95% CI 0.01 to 6.80	74%
Arrythmia	Placebo	NA	NA	NA
	Dexmedetomidine	186	OR 1.62, 95% CI 0.19 to 13.63	0%

*: Random effect model; NA: Not applicable; PONV: Postoperative nausea and vomiting.

**Table 3:** GRADE assessment of the quality of the evidence and the strength of the recommendation.

Certainty Assessment							№ of Patients		Effect		Certainty	Importance
№ of Studies	Study Design	Risk of Bias	Inconsistency	Indirectness	Imprecision	Other Considerations	[Intervention]	[Comparison]	Relative (95% CI)	Absolute (95% CI)		
VAS												
5	randomized trials	not serious	serious ^a^	not serious	Serious ^b^	publication bias strongly suspected ^c^	531	531	-	MD 0.47 lower (1.64 lower to 0.7 higher)	⨁◯◯◯ Very low	CRITICAL
Opioid consumption												
3	randomized trials	not serious	not serious	not serious	not serious	publication bias strongly suspected ^c^	244	244	-	SMD 0.34 lower (0.7 lower to 0.03 higher)	⨁⨁⨁◯ Moderate	CRITICAL
Response to verbal (time)												
6	randomized trials	not serious	not serious	not serious	not serious	publication bias strongly suspected ^c^	194	194	-	MD 1.85 higher (1.13 higher to 2.58 higher)	⨁⨁⨁◯ Moderate	IMPORTANT
Muscle relaxants consumption												
4	randomized trials	not serious	not serious	not serious	not serious	publication bias strongly suspected ^c^	154	154	-	SMD 0.91 lower (1.65 lower to 0.17 lower)	⨁⨁⨁◯ Moderate	IMPORTANT
HR												
6	randomized trials	not serious	not serious	not serious	not serious	none	514	514	-	MD 2.36 higher (0.71 lower to 5.44 higher)	⨁⨁⨁⨁ High	CRITICAL
MAP												
7	randomized trials	not serious	not serious	not serious	not serious	publication bias strongly suspected ^c^	589	589	-	MD 0.77 higher (3.64 lower to 5.17 higher)	⨁⨁⨁◯ Moderate	CRITICAL
Hypotension												
6	randomized trials	not serious	serious ^a^	not serious	not serious	none	64/198 (32.3%)	48/198 (24.2%)	OR 1.97 (1.10 to 3.52)	144 more per 1000 (from 18 more to 287 more)	⨁⨁⨁◯ Moderate	CRITICAL

^a^ The results show wide variability; ^b^ Confidence intervals are large; ^c^ Publication bias assessed by visual inspection of funnel plots; CI: confidence interval; MD: mean difference; OR: odds ratio; SMD: standardized mean difference.

## Data Availability

No new data were created or analyzed in this study. Data are contained within the article and Supplementary Materials.

## Supplementary Materials

The following supporting information can be downloaded at: https://www.mdpi.com/article/10.3390/jcm13113122/s1, Supplementary Figure S1: Publication bias assessment. Supplementary File S1: PubMed search strategy.
