# Supplementary material for: Efficacy and Safety of Intravenous Magnesium Sulfate in Spinal Surgery: A Systematic Review and Meta-Analysis
Source: J Clin Med. 2024 May 26;13(11):3122. doi: 10.3390/jcm13113122 (PMC11172721; doi:10.3390/jcm13113122)

Supplementary File 1. PubMed search strategy.

Search: **magnesium AND spine**

("magnesium"[MeSH Terms] OR "magnesium"[All Fields] OR "magnesium s"[All Fields] OR "magnesiums"[All Fields]) AND ("spine"[MeSH Terms] OR "spine"[All Fields] OR "spines"[All Fields] OR "spine s"[All Fields])

Translations

magnesium: "magnesium"[MeSH Terms] OR "magnesium"[All Fields] OR "magnesium's"[All Fields] OR "magnesiums"[All Fields]

spine: "spine"[MeSH Terms] OR "spine"[All Fields] OR "spines"[All Fields] OR "spine's"[All Fields]

Supplementary Figure 1. Publication bias assessment.

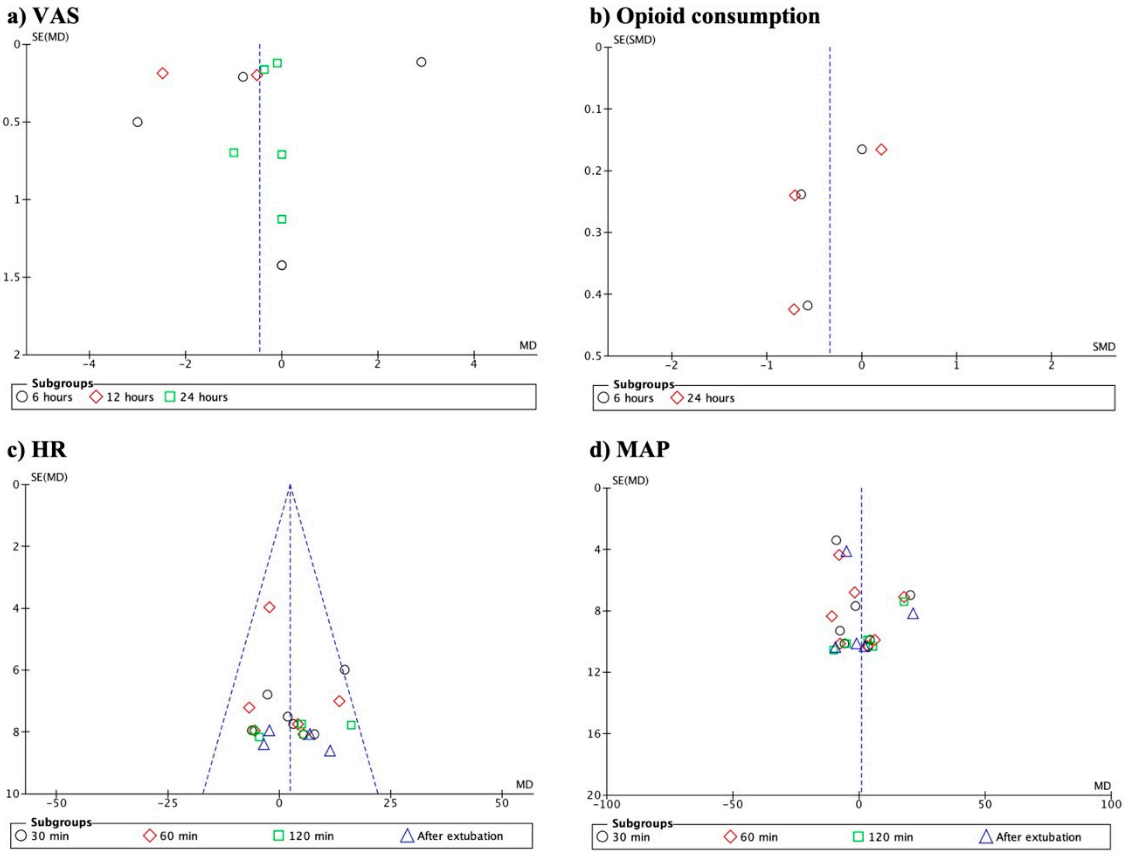

Supplement: Supplementary file 1 [file jcm-13-03122-s001.zip › jcm-3010149-supplementary.pdf]
